# U-shaped association between serum Klotho and all-cause mortality in US cardiovascular patients: a prospective cohort study

**DOI:** 10.3389/fendo.2024.1405665

**Published:** 2024-06-13

**Authors:** Shasha Liu, Zhanfang Zhu, Kai Yu, Wei Zhang, Jie Pu, Ying Lv, Zhiguo Tang, Fuqiang Liu, Yongqiang Sun

**Affiliations:** ^1^ Department of Cardiology, Shaanxi Provincial People’s Hospital, Xi’an, China; ^2^ Department of Internal Medicine, Xi’an Jiaotong University Hospital, Xi’an, China; ^3^ Department of Cardiology, Pucheng County Hospital, Weinan, China; ^4^ Department of Interventional Radiography, Shanxi Provincial People’s Hospital, Xi’an, China

**Keywords:** cardiovascular disease, serum Klotho, NHANES, all-cause mortality, National Health and Nutrition Examination Survey

## Abstract

**Background:**

Increased levels of serum Klotho have been associated with a reduced risk of several cardiovascular diseases (CVD). However, limited studies exist on the association between serum Klotho and mortality in patients with CVD.

**Methods:**

We collected data from CVD patients in the National Health and Nutrition Examination Survey (NHANES) spanning 2007 to 2016. We linked NHANES data with the National Death Index to determine the survival status of participants. Univariate and multivariable Cox regression models were used to investigate the relationship between serum Klotho levels and mortality in CVD patients. The relationship between serum Klotho quartiles and mortality in CVD patients was visualized using Kaplan-Meier (KM) curves and restricted cubic spine. Finally, subgroup analyses were used to examine the association between serum Klotho and all-cause mortality in different populations.

**Results:**

1905 patients with CVD were finally enrolled in our study with a mean follow-up of 7.1 years. The average age of the participants was 63.4 years, with 58.40% being male. KM showed that lower Klotho levels were associated with lower survival rates. After adjusting for potential confounders, patients with higher serum Klotho levels had lower all-cause mortality (Q1: 1.00, Q2: 0.58 (0.42–0.80), Q3: 0.69 (0.47–1.01), and Q4:0.64 (0.45–0.92). However, the relationship between serum Klotho levels and cardiovascular mortality was not statistically significant. Dose-response analysis shows a U-shaped relationship between serum Klotho levels and all-cause mortality in patients with CVD (P nonlinear=0.002). Subgroup analysis indicated that participants with a history of hypertension had a higher risk of all-cause mortality in serum Klotho Q4 compared to Q1 (P trend <0.05).

**Conclusion:**

The relationship between serum Klotho levels and all-cause mortality in CVD patients exhibits a U-shaped association. The underlying mechanisms of this association need further investigation.

## Introduction

Cardiovascular disease (CVD) is a major cause of global disease burden and a key factor contributing to premature death and escalating healthcare costs (1, 2). CVD encompasses a range of conditions including heart failure, coronary heart disease, angina, heart attacks, and strokes. Identifying factors that predict mortality in patients with CVD is critical to promoting early prevention of the disease.

The Klotho protein is encoded by the Klotho gene, initially described for its anti-aging abilities. Mice lacking Klotho exhibit a range of syndromes resembling human aging, including shortened lifespan, infertility, vascular calcification, arteriosclerosis, skin atrophy, osteoporosis, and emphysema (3). Conversely, overexpression of Klotho can act as an anti-aging hormone (4). Two different types of Klotho proteins can be detected in humans: one located in the cytoplasmic membrane as a transmembrane protein, and the other as soluble Klotho, including soluble and secreted Klotho circulating in the blood (5). Numerous studies have shown that increased serum Klotho levels are closely associated with decreased cardiovascular risk. In middle-aged and elderly Americans, serum Klotho levels are negatively correlated with the presence of atrial fibrillation (6). Studies in diabetic mice have found that serum Klotho levels can improve diabetic cardiomyopathy and myocardial fibrosis (7, 8). In a large and diverse cohort, an independent inverse relationship was observed between serum Klotho levels and arterial stiffness indicator, pulse pressure (9). Serum Klotho, as a cardiac protector, can prevent the effects of aging on the heart and reduce the burden of CVD (10), but there is currently no research analyzing the relationship between serum Klotho levels and all-cause mortality and cardiovascular mortality in CVD patients.

By examining data from the National Health and Nutrition Examination Survey (NHANES), this study took into account potential confounding factors and investigated the relationship between serum Klotho levels and mortality in patients with CVD. This has important implications for the management of the health of patients with CVD.

## Method

### Study population

**Figure 1:**
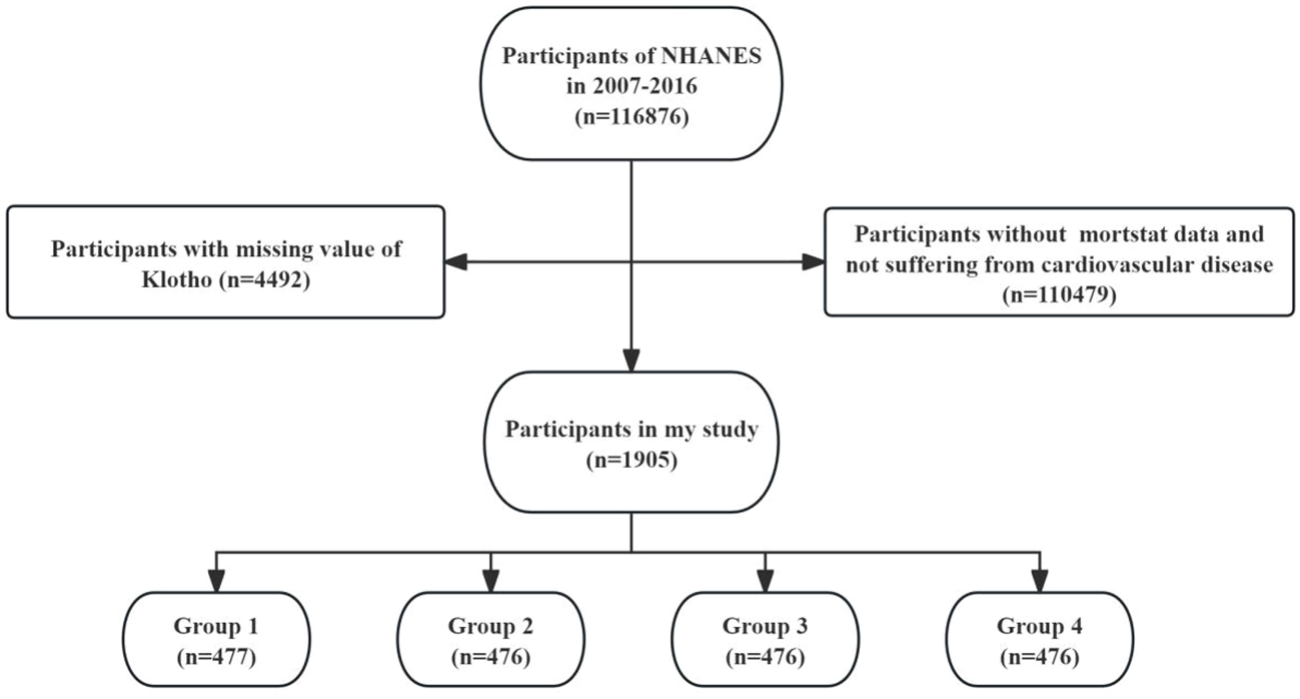
Flow chart of the study subjects.

The data used in this study were obtained from the NHANES database (https://www.cdc.gov/nchs/nhanes/index.htm). NHANES is a vital national health and nutrition survey program conducted by the U.S. government. NHANES collects a vast amount of medical, nutritional, and health-related data through population surveys and physical examinations, providing a rich source of information for research in the field of public health. In this study, we used data from 5 cycles, involving a total of 116,876 participants from 2007 to 2016. After excluding individuals with missing mortality data and without cardiovascular disease (n = 110,479), and those with missing serum Klotho data (n = 4,492), a total of 1,905 participants with CVD were included in the final analysis. The selection process of this study is illustrated in **Figure 1**.

### Outcome variable

CVD was defined as individuals who answered “yes” to the question: “Have you been told by a doctor or other health professional that you have congestive heart failure/CHD/myocardial infarction/stroke?”. All-cause mortality and cardiovascular mortality were obtained from the National Death Index (NDI) database as of December 31, 2019.

### Exposure variable

Participants’ blood samples were collected in NHANES (National Health and Nutrition Examination Survey) and stored at -80°C until analyzed. Klotho concentrations in serum were measured using a commercially available ELISA kit (product offered by IBL International, Japan). The lower limit of detection for Klotho in this kit is typically set at 6 pg/mL prior to the assay, frozen serum samples were thawed and the manufacturer’s protocol was followed. Each sample is usually assayed twice to ensure accuracy and reproducibility of results. Two concentration levels of quality control samples (low and high Klotho concentrations) were included in the assay. If a sample’s duplicate assay value varies by more than 10%, that sample is subject to re-assay. If the value of the quality control sample is outside the range of two standard deviations from the known value, the result of the entire assay panel will be considered invalid and will need to be re-assayed. The final determination is expressed as the average of two measurements (11). For a detailed description of the Klotho detection method, please visit the NHANES website.

### Covariates

We performed covariate inclusion through previous literature. Demographic, laboratory, physical examination, and questionnaire data were collected from the NHANES database. Age, poverty income ratio (PIR), and body mass index (BMI) were considered continuous variables (12, 13). Sex, race, smoking status, drinking status, education level, marital status, hypertension, and type 2 diabetes mellitus (DM) were considered categorical variables (14–16). Race was categorized as Mexican American, non-Hispanic Black, non-Hispanic White, other Hispanic, and other Race. Smoking status was categorized as former smoker, never smoker, and current smoker based on whether the participant had smoked 100 cigarettes in the past year. Drinking status was defined as non-drinker, low-to-moderate drinker, and heavy drinker. Education level was categorized as below high school, high school, and above high school. Marital status was classified as separated, married, and never married. Type 2 DM was defined according to the following criteria: (1) participants reporting a diagnosis of diabetes by a healthcare professional, (2) glycated hemoglobin testing 6.5% or higher, or (3) fasting blood glucose 126 mg/dL or higher. Hypertension was defined as self-reported hypertension, systolic blood pressure ≥140 mmHg, diastolic blood pressure ≥90 mmHg, or use of antihypertensive medication.

### Statistical analysis

Continuous variables were expressed as mean ± standard error and differences between groups were compared using weighted t-tests or one-way ANOVA. Categorical variables were expressed as frequencies and percentages, and differences between groups were compared using Chi-square tests. Univariate and multivariate Cox proportional hazards regression models were used to examine the association between serum Klotho levels and all-cause mortality in patients with CVD. In this study, we used three models: Crude model (unadjusted), Model 1 (adjusted for age, gender, and race), and Model 2 (further adjusted on the basis of model 2 for PIR, education level, BMI, hypertension, type 2 DM, drinking status, and smoking status). Kaplan-Meier (KM) curves were used to show the association between serum Klotho levels and all-cause mortality in patients with CVD when stratified by serum Klotho quartiles. In addition, restricted cubic spline curves (RCS) adjusted for confounders were utilized to show the dose-response relationship between serum Klotho levels and mortality in CVD patients. Finally, we also performed subgroup analyses and interaction tests to examine the relationship between serum Klotho levels and all-cause mortality in CVD populations with different characteristics.

All results of this study were based on clustering, stratification, and weighting calculations from the NHANES database and were analyzed using R software (version: 4.1.3) and the *survey* package (version: 4.4–1). A two-tailed p value of less than 0.05 was considered statistically significant.

## Result

### Baseline characteristics

A total of 1905 subjects were enrolled in this study, with an average age of 63.4 years, 58.40% were male, and a mean follow-up time of 7.1 years (range 0.1 to 13.2 years) (**Table 1**). Serum Klotho levels were categorized into four groups based on quartiles (Q1 ≤ 614.1, 614.1 < Q2 ≤ 763.4, 763.4 < Q3 ≤ 946.2, 946.2 < Q4, pg/mL). There were no significant differences among the four groups of participants in terms of age, sex, race, marital status, education level, BMI, PIR, smoking status, drinking status, type 2 DM, hypertension, or cardiovascular mortality (P>0.05). Participants in higher quartiles of serum Klotho exhibited a lower all-cause mortality(Q1: 30.63%, Q2:19.95%, Q3:25.35%, Q4: 20.98%, P <0.05).

**Table 1:** Baseline characteristics of participants according to quartiles of Klotho.

	Klotho quartiles (pg/mL)					P-value
	Total (N=1905)	Q1 (N=477)	Q2 (N=476)	Q3 (N=476)	Q4 (N=476)	
Age, years	63.4(0.3)	63.7(0.7)	63.8(0.5)	63.4(0.6)	62.7(0.6)	0.498
Sex (%)						0.347
female	804(41.6)	201(42.3)	185(40.1)	195(38.3)	223(46.0)	
male	1101(58.4)	276(57.8)	291(59.9)	281(61.7)	253(54.1)	
Race (%)						0.420
Mexican American	224(4.9)	55(4.9)	54(4.6)	58(5.5)	57(4.5)	
non-Hispanic Black	436(11.6)	110(12.5)	102(10.6)	108(11.2)	116(12.3)	
non-Hispanic White	918(72.2)	220(67.7)	249(75.1)	230(73.4)	219(72.4)	
other Hispanic	196(3.9)	55(4.6)	42(2.9)	46(4.1)	53(4.3)	
other Race	131(7.4)	37(10.3)	29(6.9)	34(5.9)	31(6.6)	
Marital status, %						0.209
separated	642(27.1)	167(31.8)	152(22.8)	164(27.9)	159(26.4)	
married	1113(65.7)	268(62.0)	280(67.8)	278(64.3)	287(68.5)	
never married	150(7.2)	42(6.2)	44(9.4)	34(7.8)	30(5.0)	
Education level, %						0.278
below high school	319(9.4)	66(7.6)	86(8.6)	91(12.7)	76(8.5)	
high school	816(40.8)	220(43.2)	189(38.3)	207(39.6)	200(42.3)	
above high school	767(49.8)	190(49.2)	201(53.1)	176(47.6)	200(49.3)	
BMI, kg/m^2^	31.5(0.2)	31.0(0.4)	31.6(0.4)	31.9(0.5)	31.4(0.5)	0.456
PIR	2.6(0.1)	2.4(0.1)	2.7(0.1)	2.6(0.1)	2.5(0.1)	0.182
Smoking status, %						0.100
former smoker	734(39.9)	187(39.3)	183(37.7)	194(45.9)	170(36.8)	
never smoker	688(34.8)	147(29.5)	174(38.1)	170(33.5)	197(37.7)	
current smoker	483(25.3)	143(31.2)	119(24.2)	112(20.6)	109(25.5)	
Drinking status, %						0.376
non-drinker	864(40.7)	223(47.2)	212(39.5)	213(42.6)	216(42.9)	
low-to-moderate drinker	732(43.7)	181(41.9)	179(46.3)	191(48.1)	181(47.7)	
heavy drinker	193(10.5)	45(11.0)	57(14.2)	43(9.3)	48(9.4)	
Type 2 DM, %						0.119
no	1026(59.6)	250(57.7)	287(66.1)	248(56.5)	241(57.7)	
yes	878(40.4)	227(42.3)	188(34.0)	228(43.6)	235(42.3)	
Hypertension, %						0.859
no	376(24.0)	88(22.2)	91(24.4)	102(24.1)	95(25.3)	
yes	1529(76.0)	389(77.8)	385(75.6)	374(76.0)	381(74.7)	
All-cause mortality, %						0.019
no	1355(75.9)	310(69.4)	353(80.1)	335(74.7)	357(79.0)	
yes	550(24.1)	167(30.6)	123(20.0)	141(25.4)	119(21.0)	
Cardiovascular mortality, %						0.465
no	1746(93.2)	430(91.2)	438(93.8)	437(93.4)	441(94.2)	
yes	159(6.8)	47(8.8)	38(6.2)	39(6.6)	35(5.8)	

BMI, body mass index; DM, diabetes mellitus; PIR, poverty income ratio.

All values are expressed as a proportion (%) or mean ± standard deviation.

**Table 2** stratified the participants into two groups based on their survival status, with 1355 individuals in the survival group and 55 individuals in the non-survival group. The two groups differed significantly in terms of age, marital status, education level, PIR, smoking status, drinking status, type 2 DM, hypertension, and cardiovascular mortality (P<0.05). Compared with the survivor group, the non-survivor group had a higher percentage of patients who were older, had lower PIR and education level, smoked, non-drinking, type 2 diabetes, and hypertension. In addition, cardiovascular mortality was higher in the non-survivor group.

**Table 2:** Characteristics of the study population according to their all-cause mortality.

	Total (N=1905)	Survivor (n = 1355)	Non-survivor (n = 550)	P-value
Age, years	63.4(0.3)	61.9(0.3)	68.0(0.4)	<0.001
Sex (%)				0.872
female	804(41.6)	595(41.5)	209(42.1)	
male	1101(58.4)	760(58.6)	341(57.921)	
Race (%)				0.286
Mexican American	224(4.9)	169(5.1)	55(4.2)	
non-Hispanic Black	436(11.6)	318(11.7)	118(11.1)	
non-Hispanic White	918(72.2)	604(71.1)	314(75.8)	
other Hispanic	196(3.9)	155(4.4)	41(2.7)	
other Race	131(7.4)	109(7.7)	22(6.2)	
PIR	2.6(0.1)	2.7(0.1)	2.1(0.1)	<0.001
Marital status, %				<0.001
separated	642(27.1)	421(24.2)	221(36.4)	
married	1113(65.7)	824(68.3)	289(57.6)	
never married	150(7.2)	110(7.5)	40(6.0)	
Education level, %				0.004
below high school	319(9.4)	210(8.2)	109(13.2)	
high school	816(40.8)	561(39.4)	255(45.0)	
above high school	767(49.8)	582(52.4)	185(41.8)	
BMI, kg/m^2^	31.5(0.2)	31.5(0.2)	31.2(0.6)	0.605
Klotho, pg/mL	806.6(8.6)	814.4(9.0)	782.1(16.8)	0.065
Smoking status, %				0.006
former smoker	734(39.9)	488(37.7)	246(47.0)	
never smoker	688(34.8)	526(37.0)	162(27.9)	
current smoker	483(25.3)	341(25.4)	142(25.1)	
Drinking status, %				<0.001
non-drinker	864(40.7)	571(39.4)	293(54.3)	
low-to-moderate drinker	732(43.7)	561(49.0)	171(36.5)	
heavy drinker	193(10.5)	145(11.6)	48(9.2)	
Type 2 DM, %				<0.001
no	1026(59.6)	781(63.4)	245(47.7)	
yes	878(40.4)	573(36.6)	305(52.3)	
Hypertension, %				0.033
no	376(24.0)	281(25.8)	95(18.6)	
yes	1529(76.0)	1074(74.2)	455(81.4)	

BMI, body mass index; DM, diabetes mellitus; PIR, poverty income ratio.

All values are expressed as a proportion (%) or mean ± standard deviation.

### The relationship between serum Klotho and mortality in CVD patients

**Figure 2:**
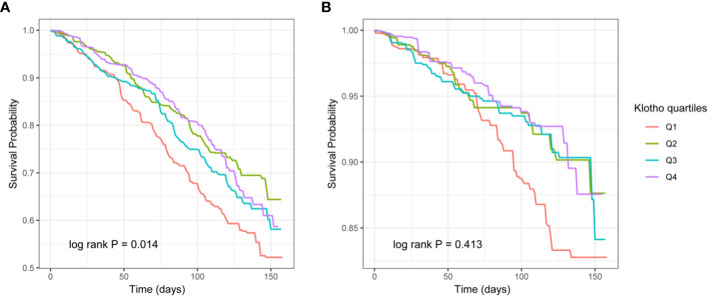
The Kaplan–Meier (KM) survival curves classified by Klotho quartiles (**A**: all-cause mortality, **B**: cardiovascular mortality).

According to the KM curve stratified by quartiles based on serum Klotho levels, the all-cause mortality in the lowest quartile (Q1) was significantly higher than in the other three groups (P = 0.014), but there was no difference in cardiovascular mortality (P >0.05) (**Figure 2**).

Further multivariate COX regression was used to analyze the independent association between serum Klotho levels and mortality (**Table 3**). In CVD patients, an inverse association was observed between serum Klotho levels and all-cause mortality across quartiles (**Table 3**). Compared with Q1, all-cause mortality was significantly lower in those with higher serum Klotho levels in fully adjusted Model 2 (Q2: 0.58 (0.42–0.80), Q3: 0.69 (0.47–1.01), and Q4: 0.64 (0.45–0.92), and the test for trend was no difference in the quartiles. However, in the study of cardiovascular mortality, no significant association was found between quartiles in the fully adjusted Model 2 (P>0.05) (**Table 4**).

**Table 3:** Association between Klotho and all-cause mortality in the CVD patients.

	Crude model		Model 1		Model 2	
	HR(95%CI)	P-value	HR(95%CI)	P-value	HR(95%CI)	P-value
Klotho quartiles						
Q1	ref		ref		ref	
Q2	0.63(0.44,0.88)	0.008	0.59(0.44,0.80)	<0.001	0.58(0.42,0.80)	<0.001
Q3	0.76(0.53,1.11)	0.158	0.75(0.52,1.09)	0.129	0.69(0.47,1.01)	0.054
Q4	0.64(0.46,0.88)	0.006	0.65(0.48,0.89)	0.007	0.64(0.45,0.92)	0.015
P for trend		0.047		0.068		0.069

Crude model: no adjusted;

Model 1: adjusted for age, sex, and race;

Model 2: adjusted for age, sex, race, PIR, education level, BMI, hypertension, type 2 DM, drinking status, and smoking status.

ref, reference; BMI, body mass index; DM, diabetes mellitus; PIR, poverty income ratio.

**Table 4:** Association between Klotho and cardiovascular mortality in the CVD patients.

	Crude model		Model 1		Model 2	
	HR(95%CI)	P-value	HR(95%CI)	P-value	HR(95%CI)	P-value
Klotho quartiles						
Q1	ref		ref		ref	
Q2	0.68(0.35,1.31)	0.246	0.63(0.34,1.18)	0.148	0.75(0.39,1.45)	0.397
Q3	0.69(0.38,1.26)	0.227	0.66(0.36,1.21)	0.178	0.66(0.33,1.35)	0.260
Q4	0.62(0.35,1.08)	0.090	0.62(0.36,1.07)	0.087	0.65(0.35,1.19)	0.158
P for trend		0.129		0.141		0.191

Crude model: no adjusted;

Model 1: adjusted for age, sex, and race;

Model 2: adjusted for age, sex, race, PIR, education level, BMI, hypertension, type 2 DM, drinking status, and smoking status.

ref, reference; BMI, body mass index; DM, diabetes mellitus; PIR, poverty income ratio.

### Dose-response relationship between serum Klotho and mortality

**Figure 3:**
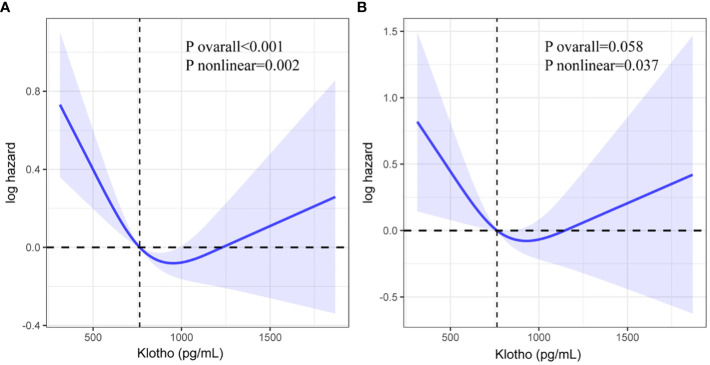
Restricted cubic spline (RCS) regression analysis of Klotho with all-cause mortality (**A**: all-cause mortality, **B**: cardiovascular mortality).

RCS was used to portray a dose-response association between serum Klotho levels and all-cause and cardiovascular mortality in patients with CVD (**Figure 3**). There was a significant nonlinear association between serum Klotho and all-cause mortality and an insignificant association with cardiovascular mortality (all-cause mortality: nonlinear P = 0.002, cardiovascular mortality: overall P>0.05). When serum Klotho levels were below 763.4 pg/mL, all-cause mortality decreased with increasing serum Klotho levels, and when Klotho was above 763.4 pg/mL, all-cause mortality increased with increasing serum Klotho levels.

### Subgroup analysis

**Figure 4:**
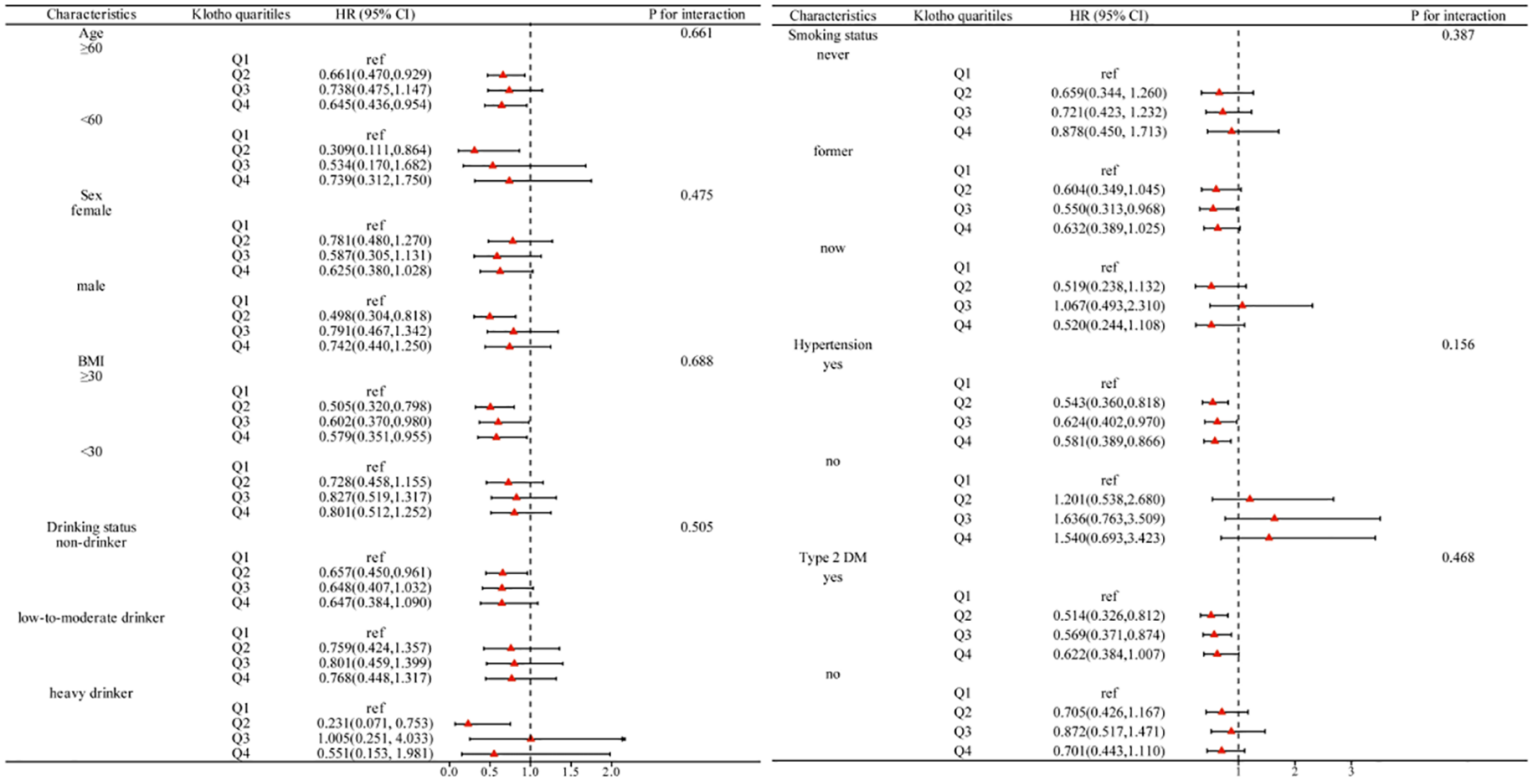
Subgroup analysis of the association between Klotho quartiles with all-cause mortality.

As depicted in **Figure 4**, a subgroup analysis was performed considering age, sex, BMI, drinking status, smoking status, hypertension, and type 2 DM. The inverse association between serum Klotho and all-cause mortality is maintained in most subgroups, and the interaction did not show differences between subgroups (P for interaction >0.05).

## Discussion

Our study represents the first investigation into the relationship between Klotho levels and mortality among CVD patients within a large population. This extensive prospective cohort study analyzed data from 1905 CVD patients included in NHANES spanning from 2007 to 2016. Upon adjusting for confounding variables, we observed that higher all-cause mortality in CVD patients was linked to lower serum Klotho levels, a finding reinforced by KM curves. Notably, serum Klotho levels did not exhibit a statistically significant association with cardiovascular mortality in CVD patients. RCS curves revealed a U-shaped correlation between serum Klotho levels and all-cause mortality in CVD patients.

Serum Klotho, an aging-related protein, is characterized by a gradual decline in levels with advancing age (17). Studies have identified its protective effects against various degenerative diseases. Notably, Serum Klotho has exhibited kidney-protective properties in animal models of acute kidney injury and chronic kidney disease (18). Nephritis mice overexpressing Klotho transgene showed improved survival rates and kidney function, along with decreased morphological alterations in renal tubules and glomeruli (19). Additionally, the kidney-protective role of serum Klotho was evident in mice with renal fibrosis, where treatment with serum Klotho protein hindered the fibrotic process (20). Furthermore, Serum Klotho has demonstrated significant involvement in degenerative lung diseases, with serum Klotho shielding the lungs from oxidative damage and cell apoptosis by enhancing the endogenous antioxidant capacity of pulmonary epithelial cells (21, 22). In the realm of cancer research, investigations have revealed an inverse relationship between serum Klotho levels and cancer risk (23). Additionally, serum Klotho has been suggested to function as a tumor suppressor gene by modulating cell metabolism and various carcinogenic pathways (24).

Experimental studies conducted on laboratory animals have highlighted the direct protective impact of serum Klotho on the cardiovascular system. Notably, Klotho-deficient mice have demonstrated arterial wall calcification (3), a risk that can be mitigated by serum Klotho supplementation resulting in decreased blood phosphate levels (25). Additionally, cardiac dysfunction, as evidenced by hypertrophy and fibrosis in Klotho-deficient mice, can be ameliorated by supplementing serum Klotho (26, 27). Clinical investigations involving patients undergoing non-emergent coronary angiography revealed an association between lower levels of serum Klotho and the presence as well as the severity of coronary artery disease (28). Furthermore, studies involving hypertensive patients showed that lower serum Klotho levels were linked to an increased risk of all-cause mortality (29).

Our findings suggest that serum Klotho levels are associated with all-cause mortality but not cardiovascular mortality in a cardiovascular disease population. Similar results were obtained in a large-scale study involving hypertensive patients, where serum Klotho levels were linked to all-cause mortality in this population but not to cardiovascular mortality (29). While two independent studies identified serum Klotho as a predictive factor for cardiovascular mortality and all-cause mortality in chronic hemodialysis patients (30, 31), it is important to note that the cardiovascular death risk in this patient population is significantly elevated compared to the general population (32).

The observed negative association between serum Klotho levels and all-cause mortality in CVD patients can be elucidated through the mechanism of oxidative stress. The Klotho protein exhibits both antioxidant and anti-apoptotic properties, thereby exerting inhibitory effects on insulin, transforming growth factor-beta 1 signaling pathways, and the release of pro-inflammatory cytokine interleukin 6. These actions serve to inhibit oxidative stress, mitigate inflammation, and prevent fibrotic effects, ultimately contributing to a reduction in the risk of all-cause mortality (20, 33–37).

Our findings reveal a U-shaped relationship between serum Klotho levels and all-cause mortality in patients with CVD, a trend that aligns with previous research. Studies involving middle-aged and older adults have similarly reported an elevated risk of death for individuals at the extremes of Klotho levels (38). Furthermore, investigations in populations with type 2 DM and rheumatoid arthritis have also identified a U-shaped association between serum Klotho levels and the risk of mortality (39, 40). Given serum Klotho’s crucial role in mineral regulation and inflammation prevention within the body (41), it is hypothesized that individuals with extreme serum Klotho levels may experience disruption in their physiological balance, subsequently increasing the risk of death.

While our research utilizing a nationally representative population provided valuable insights into the association between serum Klotho levels and the risk of death in CVD patients, it is important to acknowledge several limitations when interpreting the results. Firstly, serum Klotho levels were measured only once, and there may be other factors interfering with the accuracy of the measurement results. Secondly, the use of stored residual serum for measuring serum Klotho may introduce measurement bias stemming from sample quality issues. Thirdly, despite adjusting for confounding factors like vital signs, lifestyle habits, and comorbidities, the presence of residual confounders could potentially impact our outcomes. As a result, prospective studies are necessary to validate and confirm the findings presented in our research.

## Conclusion

Our study conducted on a nationally representative sample of American CVD patients revealed a U-shaped relationship between serum Klotho levels and all-cause mortality in this specific population. These findings offer valuable insights for prognostic assessment and treatment strategies tailored to individuals with CVD.

## Data availability statement

The raw data supporting the conclusions of this article will be made available by the authors, without undue reservation.

## Ethics statement

The studies involving humans were approved by National Center for Health Statistics Ethics Review Board. The studies were conducted in accordance with the local legislation and institutional requirements. The participants provided their written informed consent to participate in this study.

## Author contributions

SL: Writing – original draft. ZZ: Writing – original draft. KY: Writing – original draft. WZ: Writing – original draft. JP: Writing – original draft. YL: Writing – original draft. ZT: Writing – original draft. FL: Writing – original draft. YS: Writing – review & editing.
